# Supplementary material for: Quantum limit transport and destruction of the Weyl nodes in TaAs
Source: Nat Commun. 2018 Jun 7;9:2217. doi: 10.1038/s41467-018-04542-9 (PMC5992152; doi:10.1038/s41467-018-04542-9)
Supplement: Supplementary file 1 — Supplementary Information [file 41467_2018_4542_MOESM1_ESM.pdf]

Supplementary Information: Quantum Limit Transport and Destruction of the Weyl Nodes in TaAs

Ramshaw *et al.*

Supplementary Methods

A second TaAs sample (sample 2) in the **J**||**B**||**c** geometry was constructed to reproduce high field features in ρ_{zz}. This sample, shown in Supplementary Figure **3**, is actually the same as shown in Supplementary Figure **1** but with the current path even more restricted.

The ρ_{zz} data in Supplementary Figure **3** is qualitatively similar to that shown in Figure 1 of the main text (sample 1): both show the quantum limit of Weyl pockets near 7.5 tesla, both have a nearly field and temperature independent resistivity beyond the ultra quantum limit of the hole pocket at 36 tesla, and both show the onset of increased resistivity in the 50 to 60 tesla range (depending on temperature). The difference near the ultra-quantum limit could be due to different mobilities between sample 1 and sample 2, or due to a slight misalignment in the **J**||**B**||**c** geometry. Because the hole pocket has an aspect ratio of more than 10:1, a $15°$ offset can produce a large change in curvature factor (easily a factor of 3 for a mass anisotropy of 10 [1]) and hence a large change in oscillation amplitude from this pocket. Nevertheless the conductivity above the quantum limit, and before the gap opens above 50 tesla, is roughly field-independent.

Figure **4** shows a sample prepared by plasma FIB, with the field applied approximately 25 degrees off from the $\hat{c}$-axis (based on the positions of the quantum oscillations and the angle dependence of Arnold et al. [2]). This pushes the quantum limit of the Weyl pockets up to around 8.5 tesla from 7.5 tesla, the ultra-quantum limit of the hole pocket up to approximately 40 tesla from 36 tesla, and the onset of the gap to 60 tesla from 50 tesla. This increase in the onset field as the field is rotated toward the plane in which the Weyl nodes are separated was predicted by Kim et al. [3].

The correct resistivity scaling of c1 and c2 below the gap opening at around 60 tesla—despite their very different aspet ratios—indicates that the transport in this regime is bulk-dominated and that current jetting has been eliminated. The breakdown of the correct scaling once the gap opens suggests that the high-field transport becomes dominated by the surface conduction once the bulk becomes insulating.

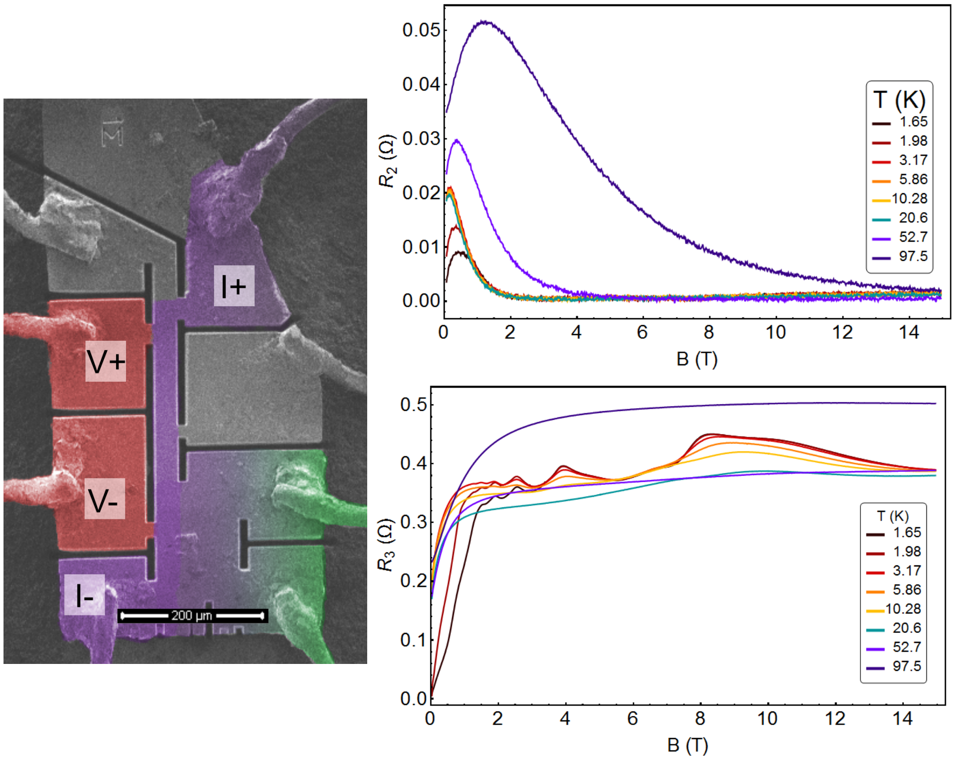

Supplementary Figure 1. **Demonstration of geometry-induced negative magnetoresistance in TaAs.** *Left:* Single crystal TaAs prepared through focused ion beam lithography to demonstrate the effects of inhomogeneous current paths. The current is injected along the purple path: the voltage is measured at the red contacts—near the current path—and at the green contacts—away from the current path. The crystalline $\hat{c}$-axis points along the current path: we applied B along this direction as well. *Upper right:* Apparent resistance measured at the contact pads shaded in green. A large negative magnetoresistance is observed at low fields: at high fields no voltage drop is observable due to the fact that no current travels near the green contacts. *Bottom right:* Contact pads placed along the current path reveal the "intrinsic" resistance of the material: roughly constant background magnetoresistance with quantum oscillations above 1 tesla, where $\omega_c\tau \approx 1$.

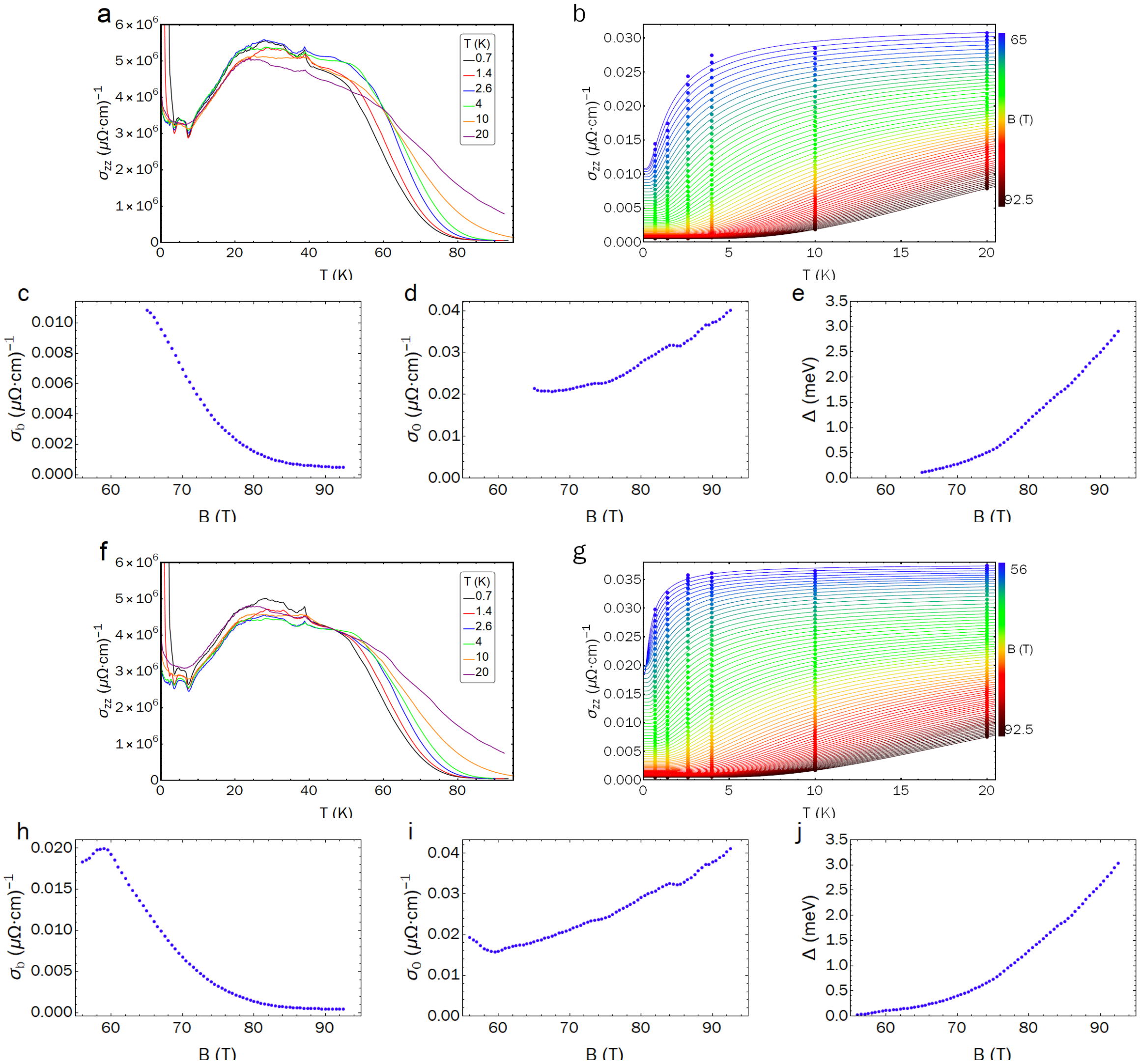

Supplementary Figure 2. Fits to σ_{zz} as a function of temperature for different magnetic field values. a, Raw conductivity data: the feature near 40 tesla is a voltage spike induced when the capacitor bank is discharged into the insert magnet. **b**, The conductivity in **a** is fit to $\sigma_{zz} = \sigma_0 + \sigma_1 e^{-\frac{\Delta}{k_B T}}$, with fit parameters shown in **c**, **d**, and **e**. Fits below 65 T are impossible due to a temperature-dependent background. To alleviate this we normalized the conductivity to the 20 kelvin value at 47 tesla (**f**), with the resultant fit shown in **g** and parameters in **h**, **i**, and **j**. Note that Δ is largely independent of this procedure.

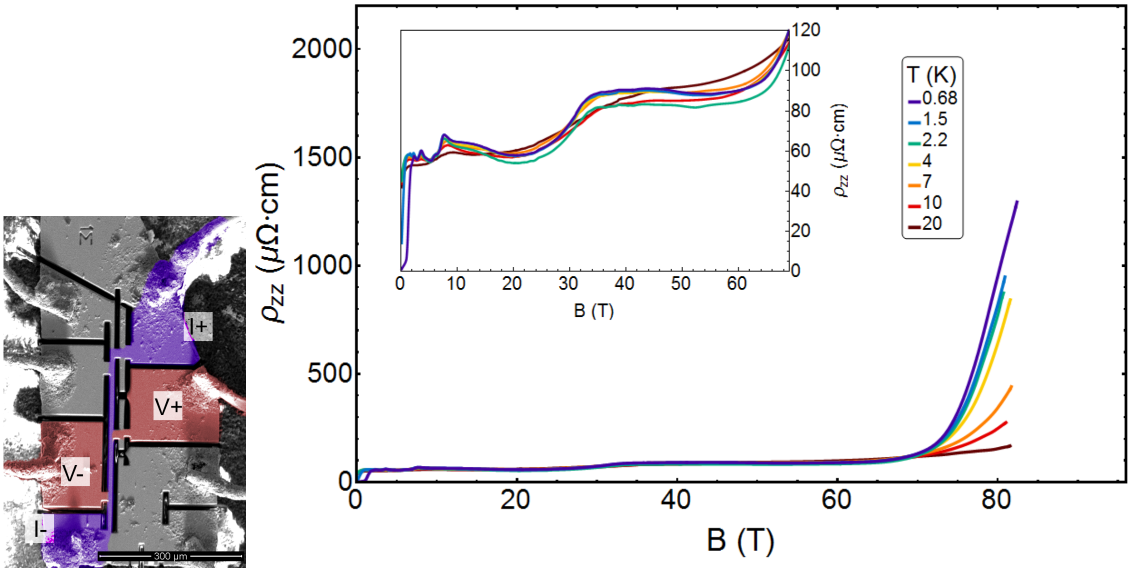

Supplementary Figure 3. $J||B$ **resistivity for TaAs sample number 2.** *Left:* the same sample shown in Supplementary Figure **1** but with the current path (purple) restricted further, and voltage was measured between the red contacts. *Right:* Resistivity up to 82 tesla from 0.68 to 20 kelvin. Most features are qualitatively similar to those in Figure 1 of the main text, baring a more pronounced feature at the ultra-quantum limit of the hole pocket near 36 tesla (the ultra quantum limit being the field where the $n = 0$ LL is depopulated due to a fixed chemical potential).

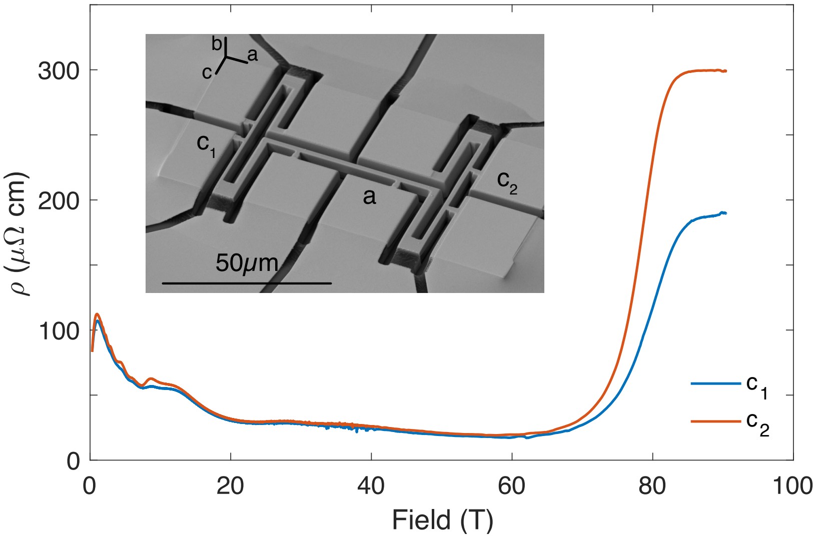

Supplementary Figure 4. ***c*-axis resistivity for TaAs sample number 3.** This sample (inset) was prepared with a xenon FIB, in contrast with the gallium FIB used for the other devices. Resistivity sections c1 and c2 were cut with different aspect ratios to probe surface conductance effects (dimensions of c1 are $h = 3.4\ \mu\text{m}$, $w = 1.25\ \mu\text{m}$, and $l = 7.5\ \mu\text{m}$; dimensions of c2 are $h = 3.4\ \mu\text{m}$, $w = 1.8\ \mu\text{m}$, and $l = 16\ \mu\text{m}$). The resistivity for both legs scales as bulk resistivity up to 60 tesla, at which point surface conductance starts to overwhelm the bulk.

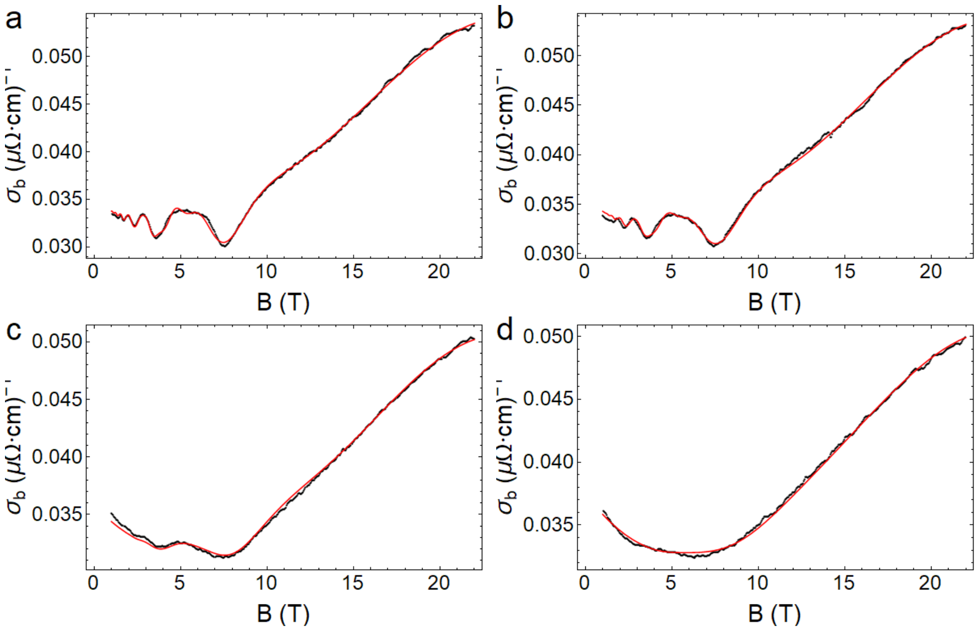

Supplementary Figure 5. **Fits to the oscillatory conductivity.** The conductivity for the sample discussed in the main text at 2.6 (**a**), 4 (**b**), 10 (**c**), and 20 (**d**) kelvin. Data is in black, fits to Eq. 3 of the supplementary are in red. One fit is performed to all 4 data sets at once to constrain the fit parameters. A third order polynomial in temperature and field is used to fit the background.

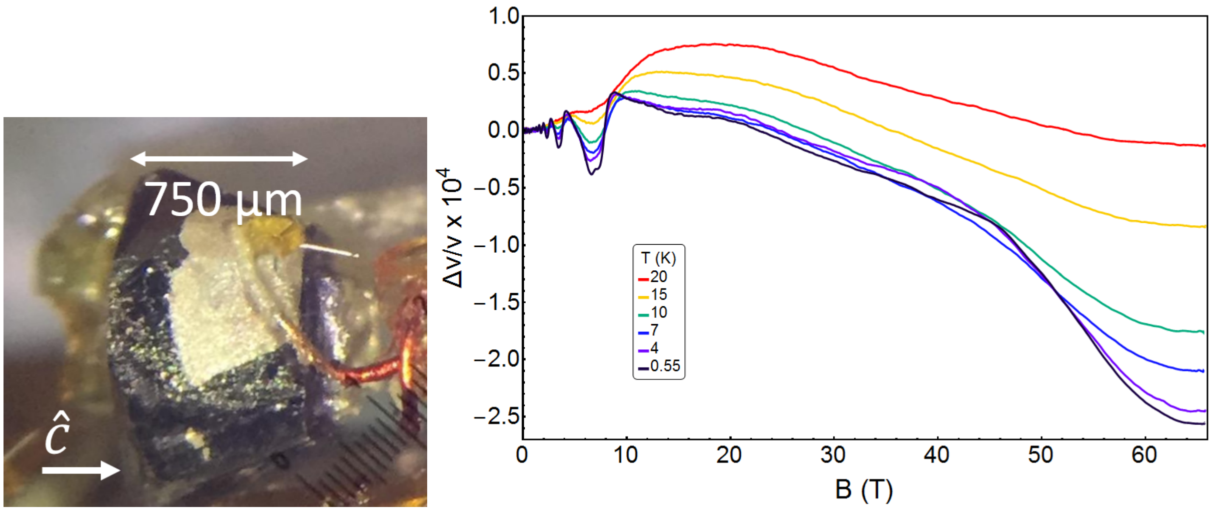

Supplementary Figure 6. **TaAs sample and ultrasound data from 0 to 65 tesla.** *Left:* TaAs sample used for ultrasound. Two parallel faces approximately $500 \times 500\ \mu m^2$ were polished perpendicular to the $\hat{c}$ axis. The sample was grounded with silver paint since the backside of the $LiNbO_3$ transducer was inaccessible. *Right:* Change in sound velocity from 0.55 to 20 K, from 0 to 65 tesla.

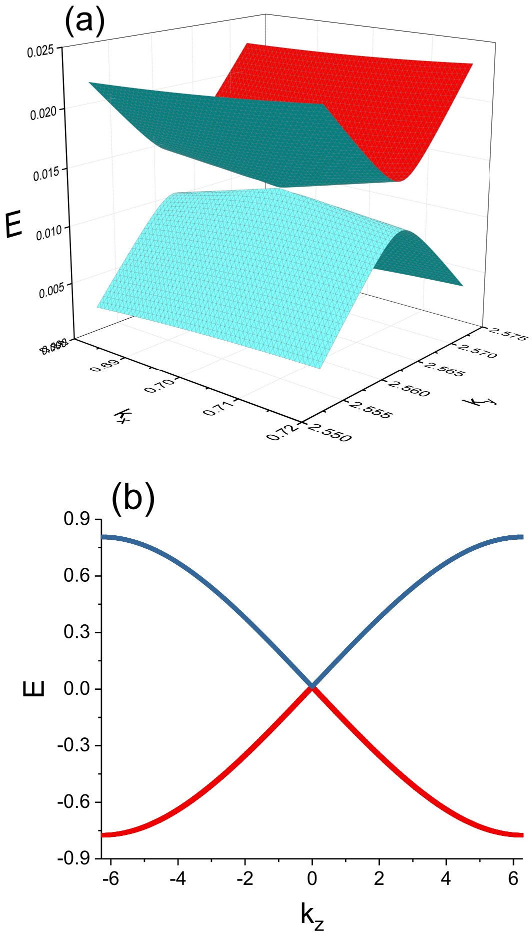

Supplementary Figure 7. The dispersion of the tight binding model in Eq. **??** in the (a) $k_x - k_y$ plane and (b) k_z direction around $\vec{k} = (0.7, 2.56, 0)$ shows that the gap closes at a single Weyl node and the nearby dispersion is linear. The model parameters are $t = 1.0$, $\Delta = 0.7$, and $\lambda = 0.1$.

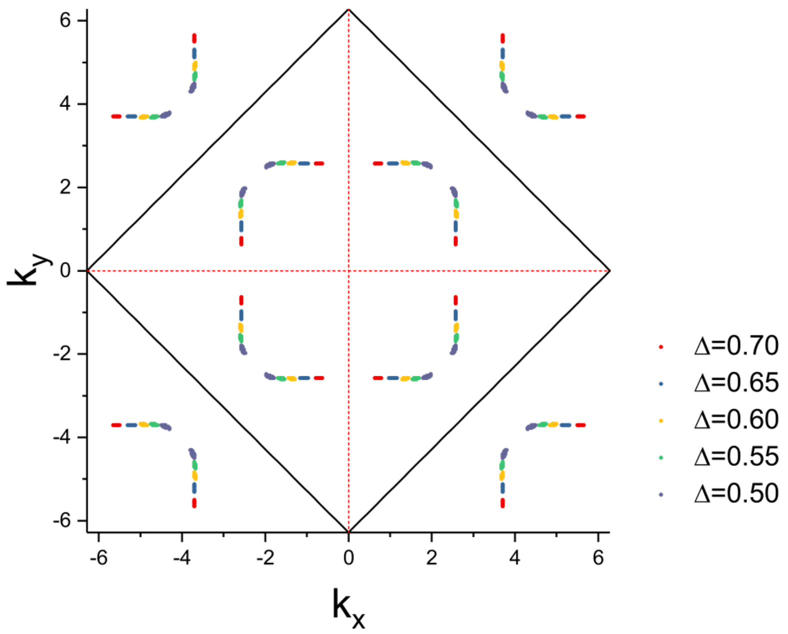

Supplementary Figure 8. The locations of the eight Weyl points in the $k_x - k_y$ plane at $k_z = 0$. The data points reflects the regions where the band gap falls below $0.03t$. The solid black lines mark the boundary of the Brillouin zone and the red dashed lines are the mirror symmetry planes. As Δ increases, the eight Weyl points approach each other and finally annihilate in pairs on the mirror planes at $\Delta \sim 0.8$, leading to a trivial insulator. Other parameters are $t = 1.0$, $\delta = 0.7$ and $\lambda = 0.1$.

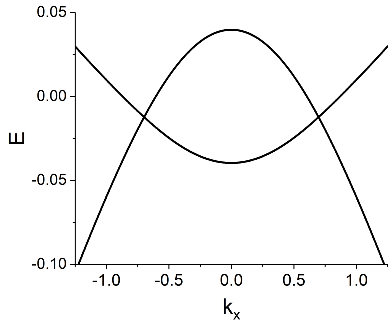

Supplementary Figure 9. The dispersion along the pair of adjacent Weyl nodes, from our tight-binding model with parameters $t = 1.0$, $\delta = 0.7$ and $\lambda = 0.1$ for illustration. This dispersion is similar to that obtained via DFT calculations by Ma et al. [4] along a cut through the W2 Weyl nodes.

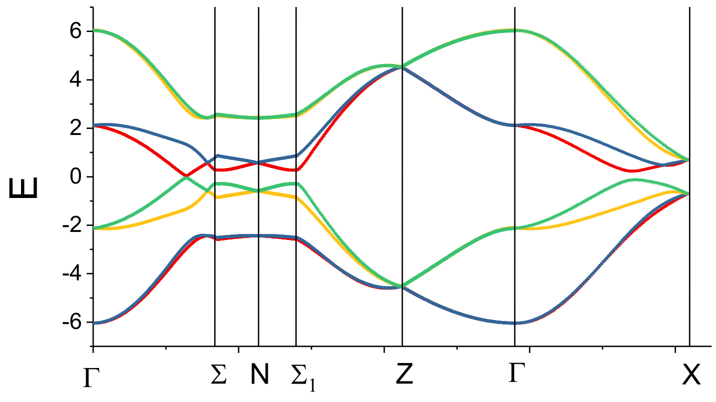

Supplementary Figure 10. The bulk electronic structure of (upper) the tight-binding model in Eq. **??** and (lower)—the tight-binding model parameters are $t = 1.0$, $\Delta = 0.7$, and $\lambda = 0.1$. The general structure compares favorably with electronic structure calculations from Ref. [5]

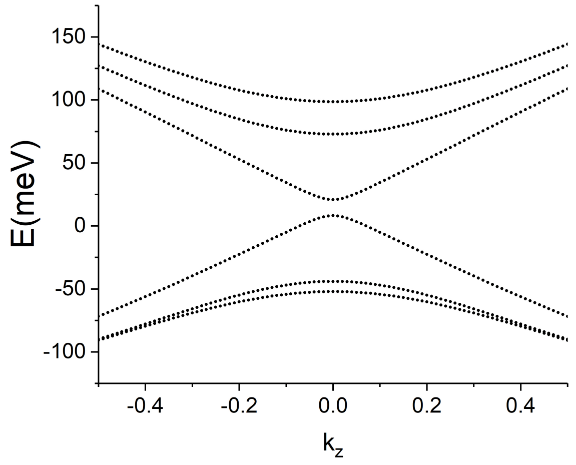

Supplementary Figure 11. Landau band dispersion along the k_z direction obtained for the tight-binding model in Eq. 7 of the methods. $t = 1.0$, $\Delta = 0.7$, and $\lambda = 0.1$. The vector potential A applied is equivalent to the W2 Weyl nodes in the presence of a magnetic field $B_z = 145T$. Each band is four-fold degenerate. The bands at higher and lower energies are the non-chiral Landau bands at indices $n = \pm 1, \pm 2$.

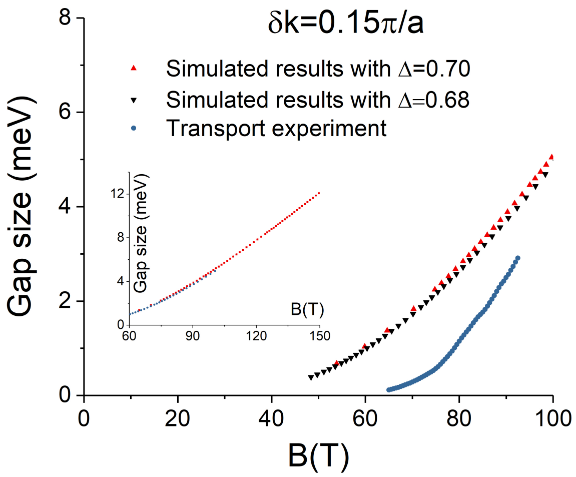

Supplementary Figure 12. The size of the gap between the $n = 0$ chiral Landau bands versus the magnetic field. Two numerical simulations are carried out with $\Delta = 0.68$ and $\Delta = 0.70$, respectively. $t = 1$ and $\lambda = 0.1$ for both. The low-energy physics around the pairs of Weyl nodes is compared with experimentally-measured properties for the W2 Weyl nodes in TaAs: $v_F^x \sim v_F^y \sim 1.92 eV \cdot \mathring{A}$ and $\delta k \sim 0.15\pi/a$. The values of the gap extracted from the temperature dependence in transport experiments are also listed for comparison. Inset: at larger magnetic field the gap shows a linear dependence.

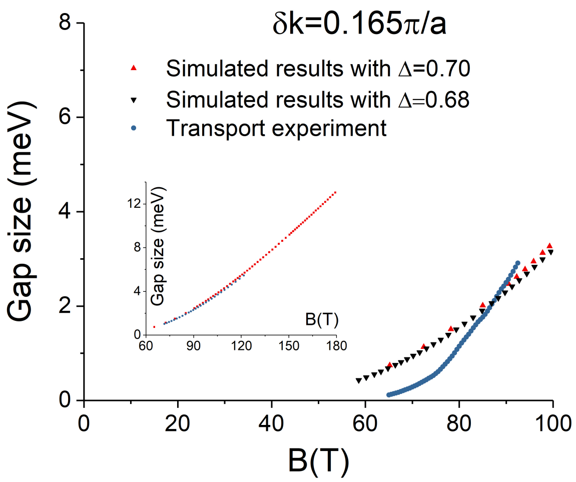

Supplementary Figure 13. Same as Fig. **12**, except the separation between the Weyl nodes in TaAs is assumed to be $\delta k \sim 0.165\pi/a$. Note the sensitive dependence of the gap size on δk.

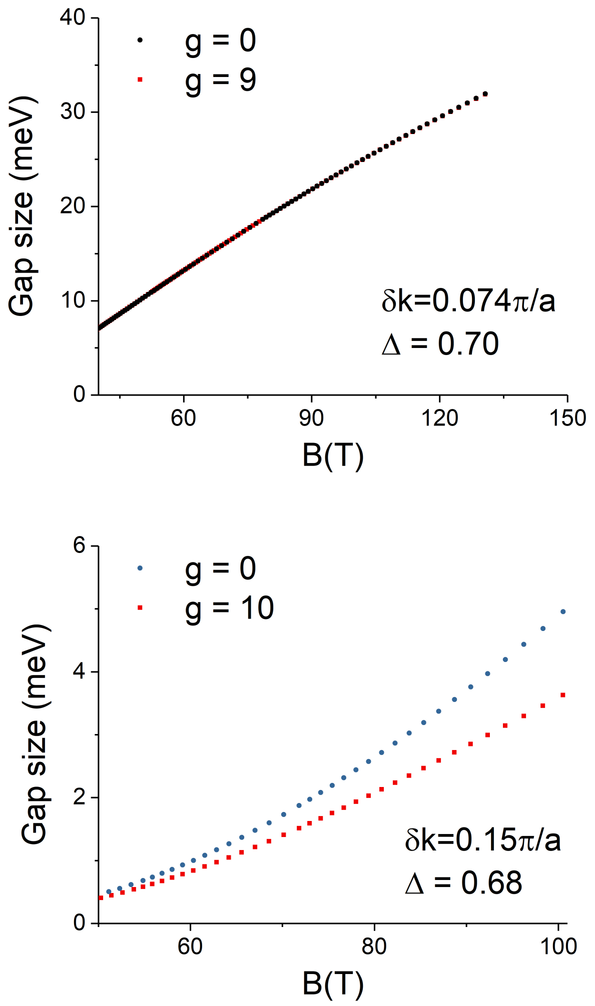

Supplementary Figure 14. Parallel comparisons of the magnetic field dependence of the gap opened between chiral Landau levels with and without the Zeeman effect incorporated as Eq. 8 in the supplementary. Upper: $\Delta = 0.7$ in the tight-binding model and the data is interpreted for Weyl nodes separation $\delta k = 0.074\pi/a$; Lower: $\Delta = 0.68$ and $\delta k = 0.15\pi/a$ where smaller gaps make the Zeeman effect's impact more apparent. Both have $t = 1$ and $\lambda = 0.1$ as tight-binding mode parameters.

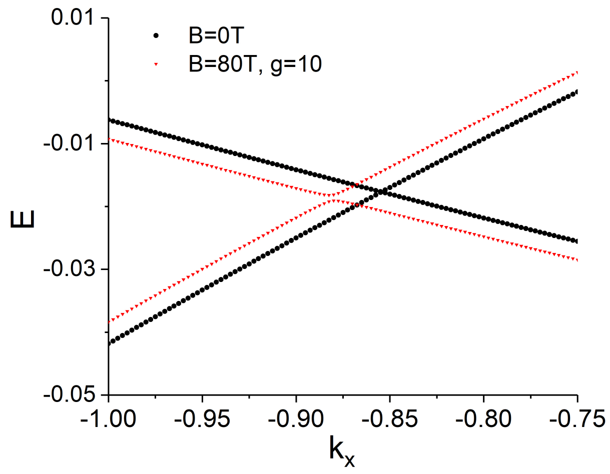

Supplementary Figure 15. The dispersion near a Weyl nodes along the direction to its pair counterpart without and with a Zeeman energy equivalent to 80T of magnetic field and a g-factor of 10. In the presence of the Zeeman energy, the Weyl nodes separation increases.

Supplementary References

[1] D. Shoenberg. *Magnetic oscillations in metals*. Cambridge monographs on physics. Cambridge University Press, 1984. ISBN 9780521224802.

[2] F. Arnold, M. Naumann, S.-C. Wu, Y. Sun, M. Schmidt, H. Borrmann, C. Felser, B. Yan, and E. Hassinger. Chiral Weyl Pockets and Fermi Surface Topology of the Weyl Semimetal TaAs. *Phys. Rev. Lett.*, 117:146401, Sep 2016. doi:10.1103/PhysRevLett.117.146401. URL https://link.aps.org/doi/10.1103/PhysRevLett.117.146401.

[3] Pilkwang Kim, Ji Hoon Ryoo, and Cheol-Hwan Park. Breakdown of the Chiral Anomaly in Weyl Semimetals in a Strong Magnetic Field. *Physical review letters*, 119(26):266401, 2017.

[4] Qiong Ma, Su-Yang Xu, Ching-Kit Chan, Cheng-Long Zhang, Guoqing Chang, Yuxuan Lin, Weiwei Xie, Tomás Palacios, Hsin Lin, Shuang Jia, et al. Direct optical detection of Weyl fermion chirality in a topological semimetal. *Nature Physics*, 13(9):842, 2017.

[5] Xiaochun Huang, Lingxiao Zhao, Yujia Long, Peipei Wang, Dong Chen, Zhanhai Yang, Hui Liang, Mianqi Xue, Hongming Weng, Zhong Fang, Xi Dai, and Genfu Chen. Observation of the Chiral-Anomaly-Induced Negative Magnetoresistance in 3D Weyl Semimetal TaAs. *Phys. Rev. X*, 5: 031023, Aug 2015. doi:10.1103/PhysRevX.5.031023. URL http://link.aps.org/doi/10.1103/PhysRevX.5.031023.
