# Supplementary material for: Quantum limit transport and destruction of the Weyl nodes in TaAs
Source: Nat Commun. 2018 Jun 7;9:2217. doi: 10.1038/s41467-018-04542-9 (PMC5992152; doi:10.1038/s41467-018-04542-9)
Supplement: Supplementary file 2 — Peer Review File [file 41467_2018_4542_MOESM2_ESM.pdf]

Editorial Note: this manuscript has been previously reviewed at another journal that is not operating a transparent peer review scheme. This document only contains reviewer comments and rebuttal letters for versions considered at Nature Communications.

REVIEWERS' COMMENTS:

Reviewer #1 (Remarks to the Author):

After a careful revision, I recommend publication of this manuscript in Nature Communications.

The authors have addressed all comments from the referees convincingly.
